# Supplementary material for: Biological Evaluations, NMR Analyses, Molecular Modeling Studies, and Overview of the Synthesis of the Marine Natural Product (−)-Mucosin
Source: Molecules. 2024 Feb 24;29(5):994. doi: 10.3390/molecules29050994 (PMC10933799; doi:10.3390/molecules29050994)
Supplement: Supplementary file 1 [file molecules-29-00994-s001.zip › molecules-2880634-supplementary.pdf]

Biological Evaluations, NMR Analyses, Molecular Modelling Studies, and Overview of the Synthesis of the Marine Natural Product (–)-Mucosin

Jens M. J. Nolsøe[1,6], Jarl Underhaug[2], Åshild Moi Sørskar,[3] Simen Gjelseth Antonsen[4], Karl E. Malterud[3], Osman Gani,[3] Qiong Fan,[5] Marit Hjorth,[5] Thomas Sæther[5], Trond V. Hansen[1,3], and Yngve H. Stenstrøm[1,4]*

[1] Faculty of Chemistry, Biotechnology and Food Science, Norwegian University of Life Sciences, P.O. Box 5003, NO-1433 Ås, Norway; yngve.stenstrom@nmbu.no (Y.H.S.); t.v.hansen@farmasi.uio.no (T.V.H.), jens.m.nolsoe@nord.no (J.M.J.N.).

[2] Department of Chemistry, University of Bergen, Allégaten 41, NO-5007, Bergen, Norway; jarl.underhaug@uib.no (J.U.).

[3] Department of Pharmacy, Section for Pharmaceutical Chemistry, University of Oslo, P.O. Box NO-1068, 0316 Oslo, Norway; a.m.sorskar@farmasi.uio.no (Å.M.S.); k.e.malterud@farmasi.uio.no (K.E.M.); osman.gani@farmasi.uio.no (O.M.); t.v.hansen@farmasi.uio.no (T.V.H.).

[4] Department of Mechanical, Electronic and Chemical Engineering, Faculty of Technology, Art and Design, Oslo Metropolitan University, NO-0130 Oslo, Norway; simenant@oslomet.no (S.G.A.).

[5] Department of Molecular Medicine, Institute of Basic Medical Sciences, Faculty of Medicine, University of Oslo, NO-0317 Oslo, Norway; qiong.fan@medisin.uio.no (Q.F.); marit.hjorth@medisin.uio.no (M.H.); thomas.sather@medisin.uio.no (T.S.).

[6] Faculty of Biosciences and Aquaculture, Nord University, P.O. Box NO-1490, 8049 Bodø, Norway; jens.m.nolsoe@nord.no (J.M.J.N.).

- Correspondence: yngve.stenstrom@nmbu.no; Tel.: +47-67232559 (Y.H.S.).

Innhold

General Information..1

NMR-data..2

General Information

The stated yields are based on isolated material.

The NMR spectra were acquired at 298 K on an 850 MHz Bruker AVANCE III HD equipped with a TCI CryoProbe (Bruker BioSpin, Billerica, MA, USA). Coupling constants (*J*) are reported in hertz and chemical shifts (δ) are reported in parts per million (ppm), referenced to the residual solvent signal (7.26 ppm for ^{1}H and 77.16 ppm for ^{13}C).

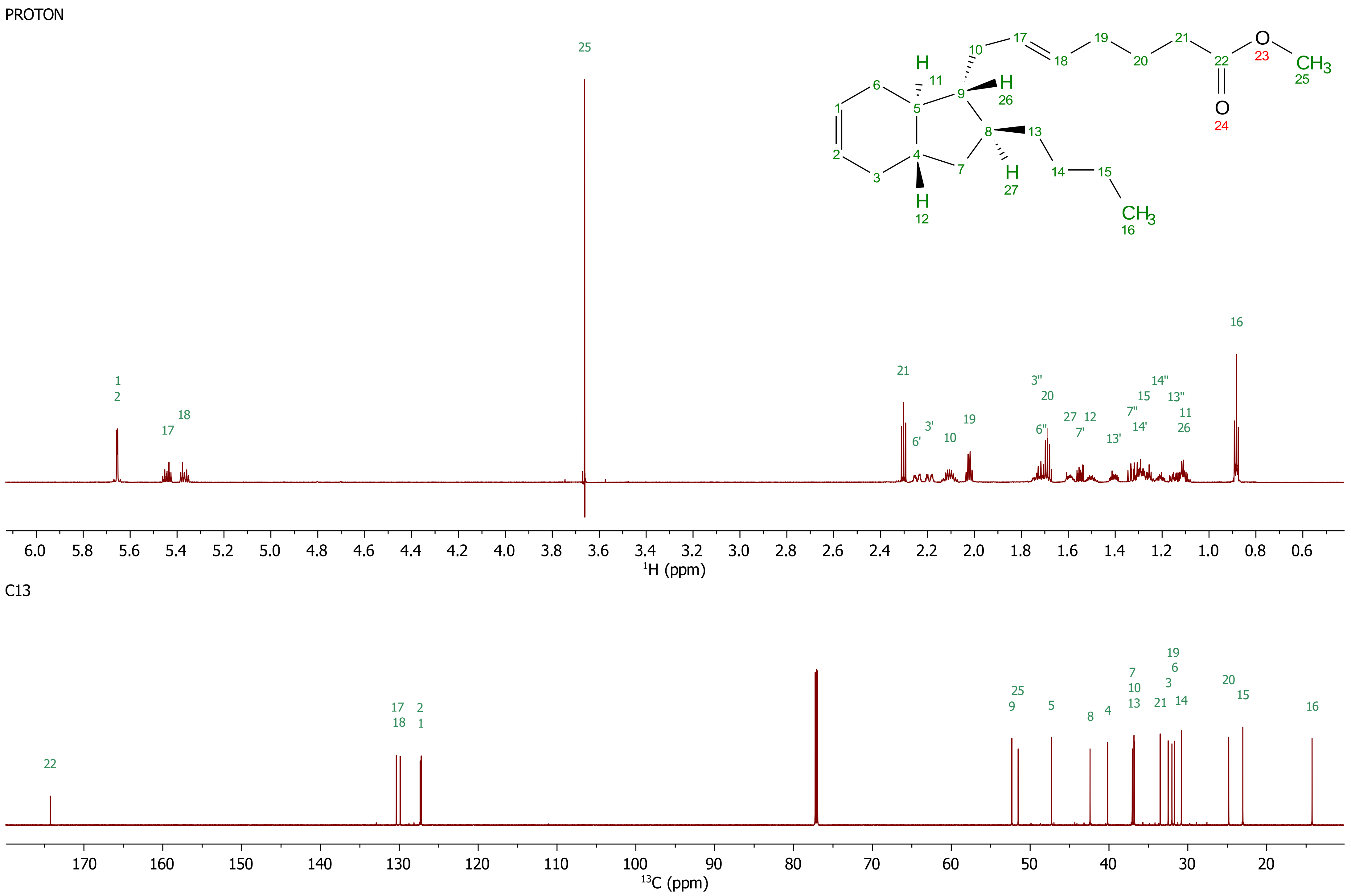

Figure S1: ^{1}H and ^{13}C NMR of **7** with assignment

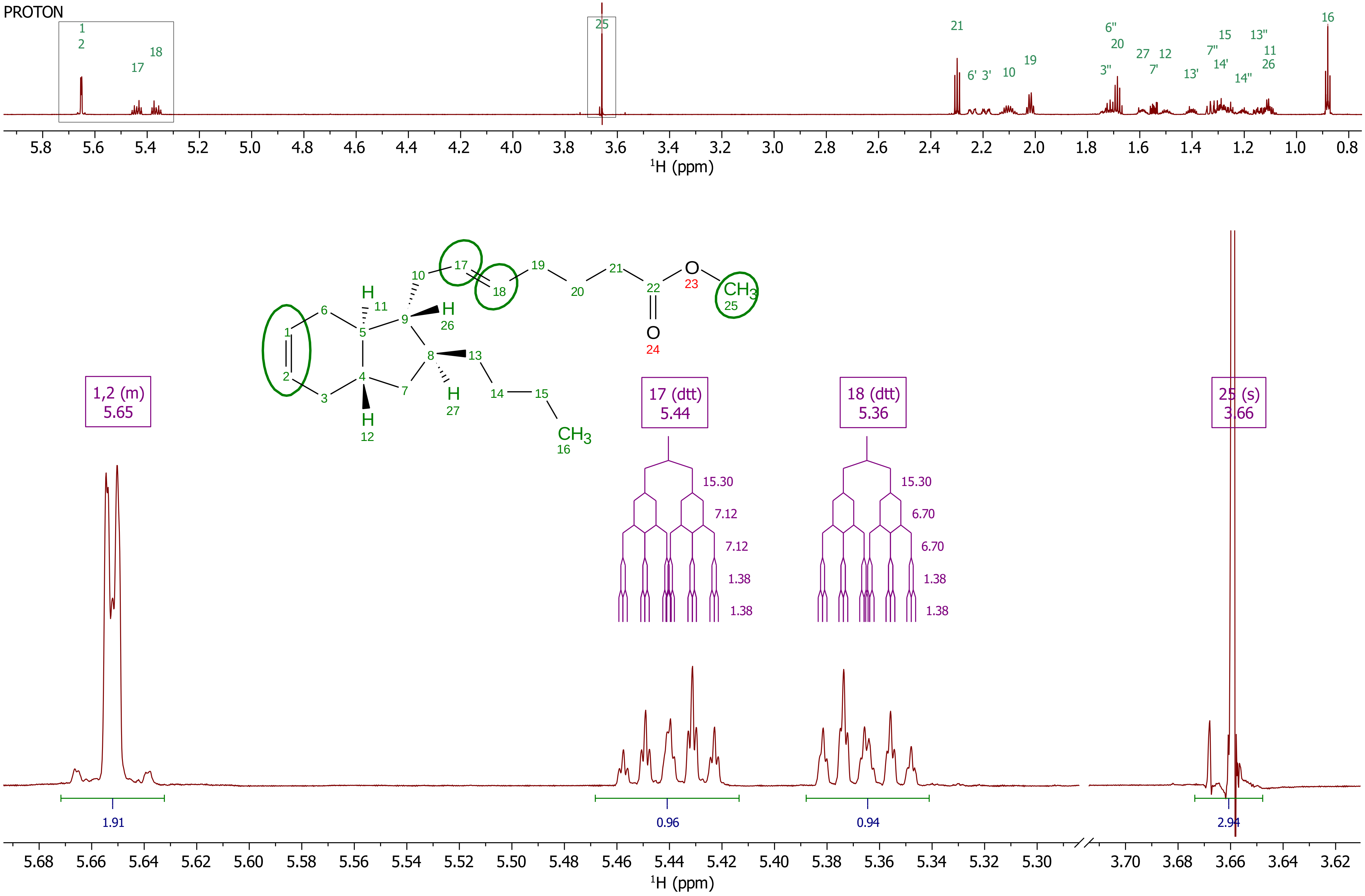

Figure S2: Extract of ^{1}H NMR of **7** with assignment

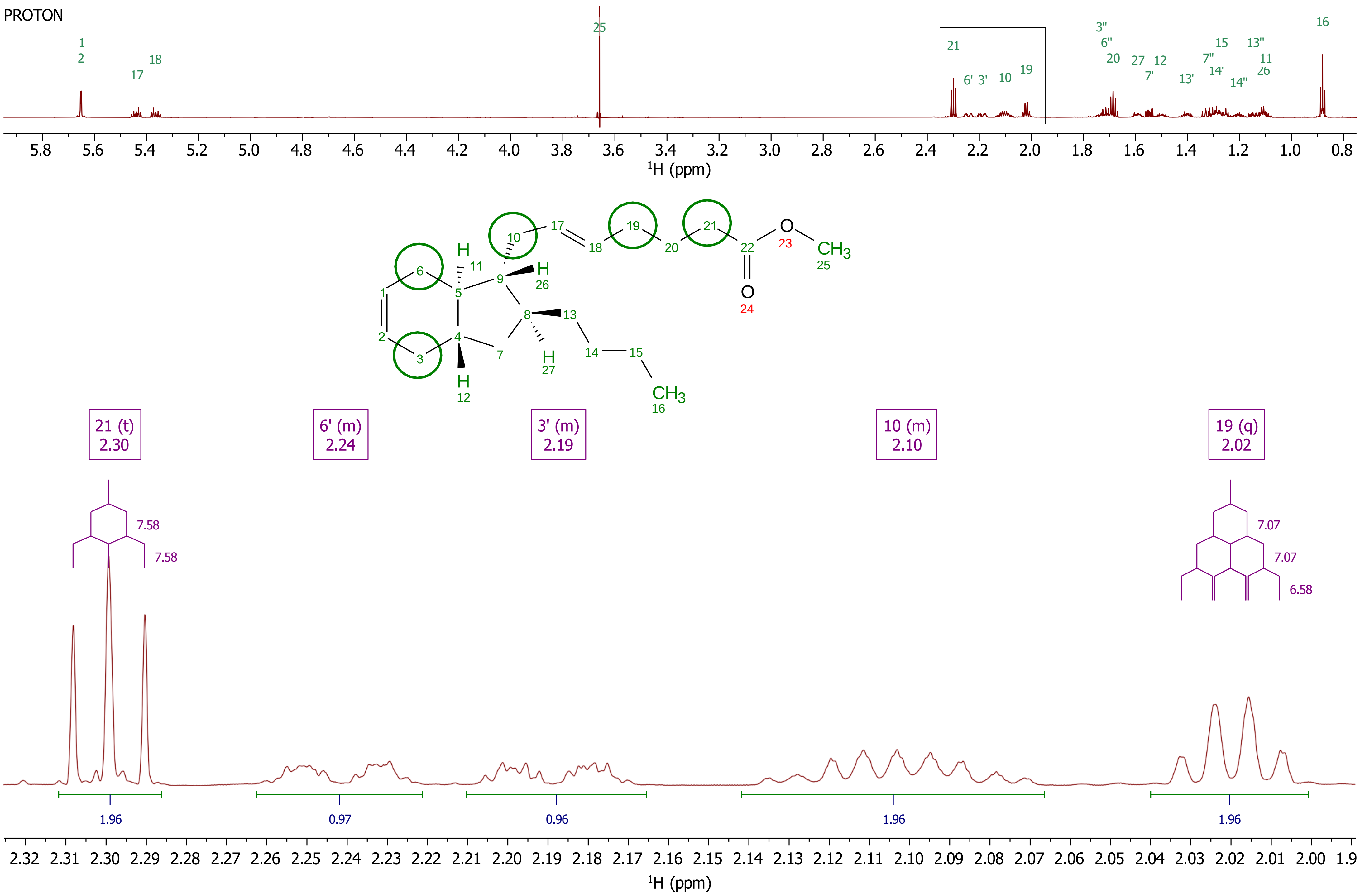

Figure S3: Extract of ^{1}H NMR of **7** with assignment

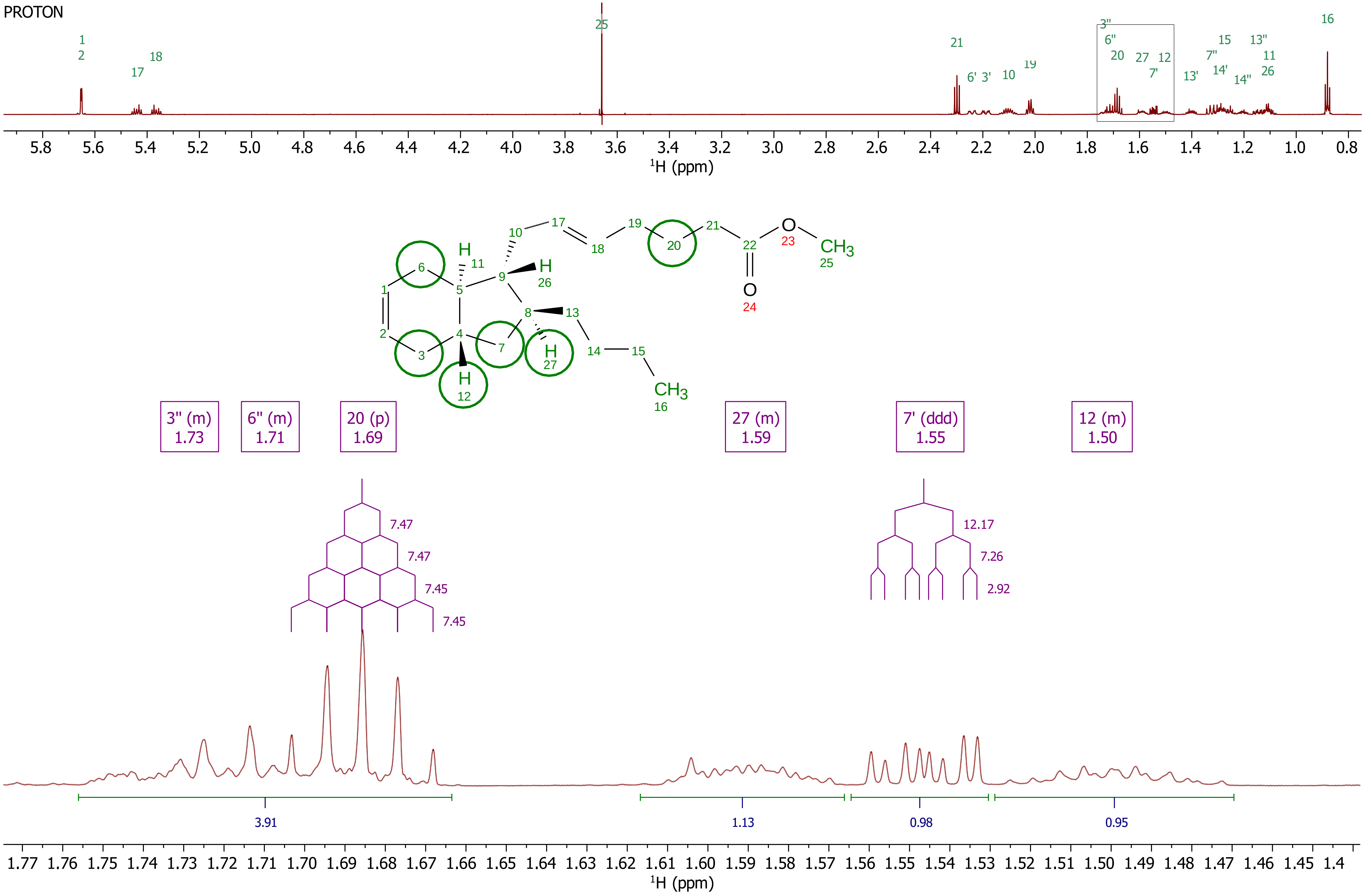

Figure S4: Extract of ^{1}H NMR of **7** with assignment

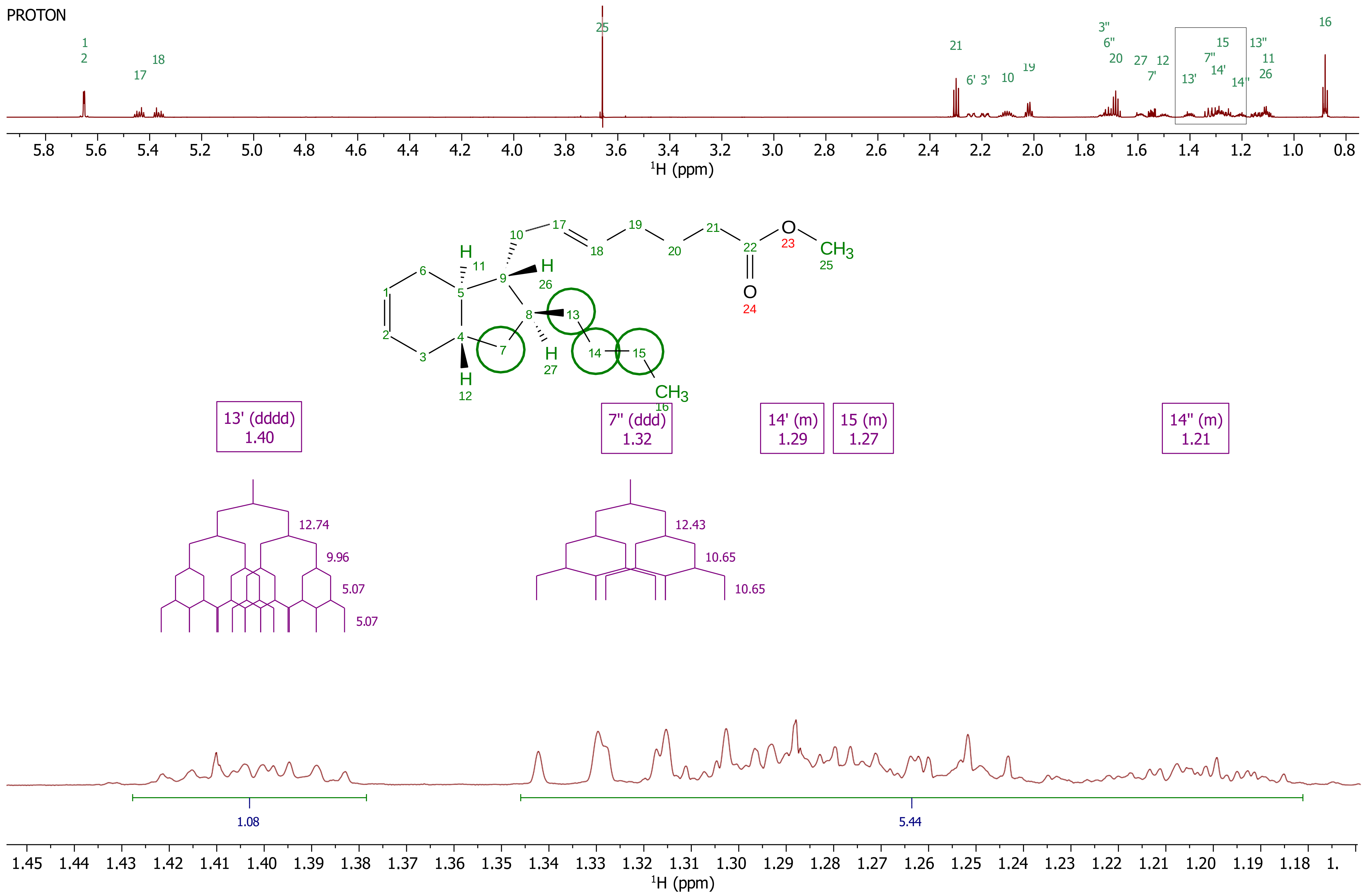

Figure S5: Extract of ^{1}H NMR of **7** with assignment

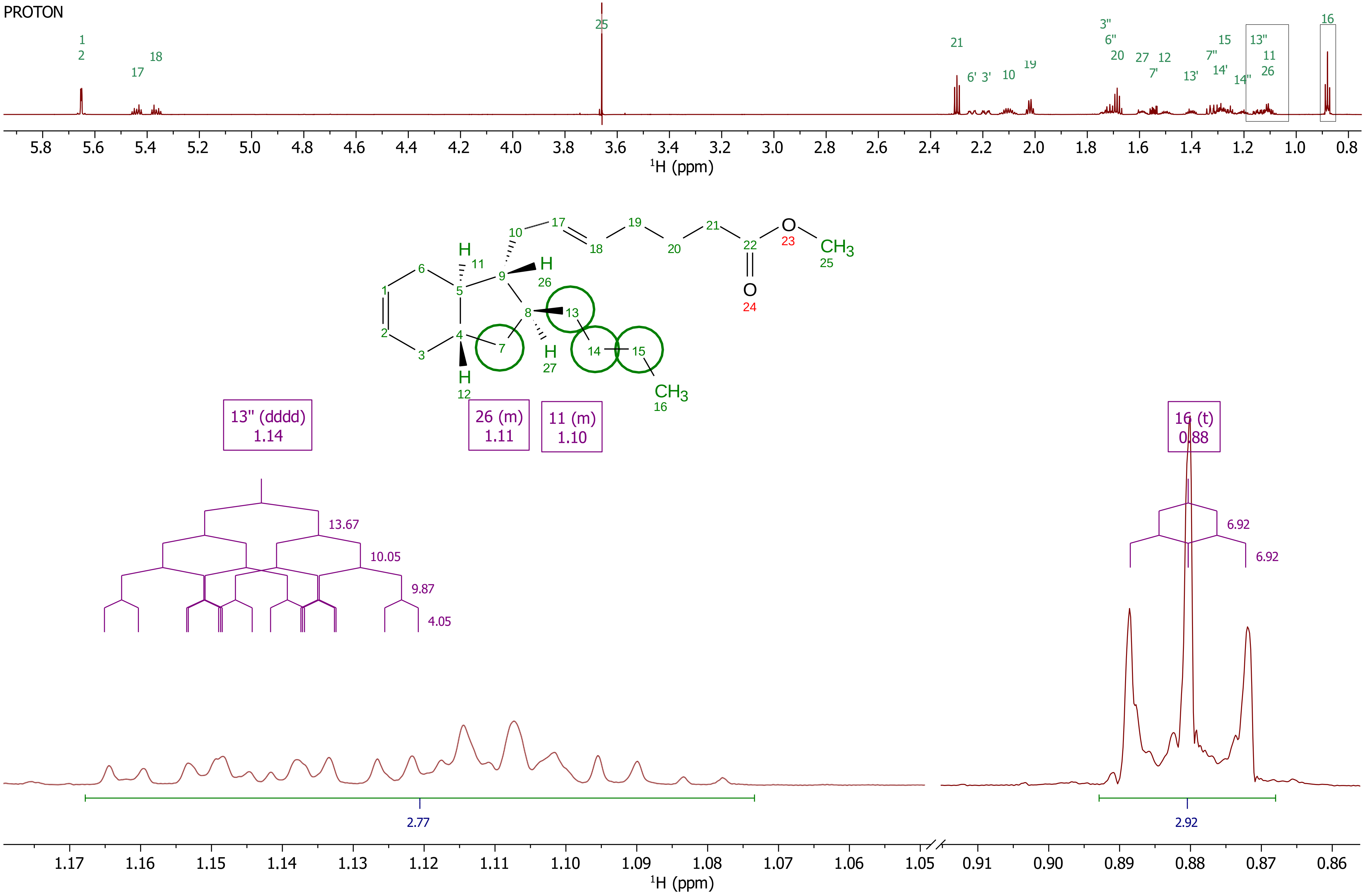

Figure S6: Extract of ^{1}H NMR of **7** with assignment

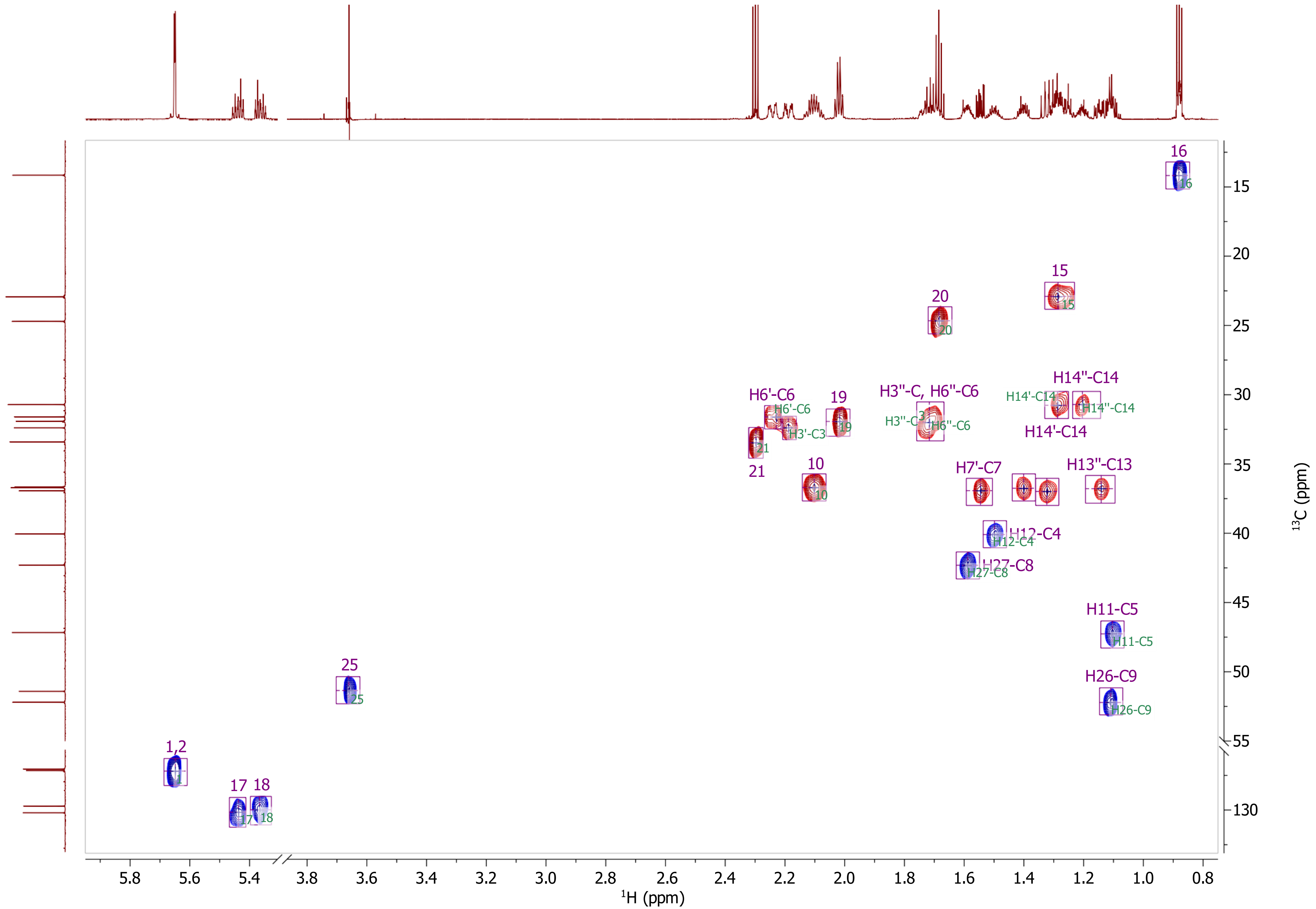

Figure S7: HSQC of **7** with assignment

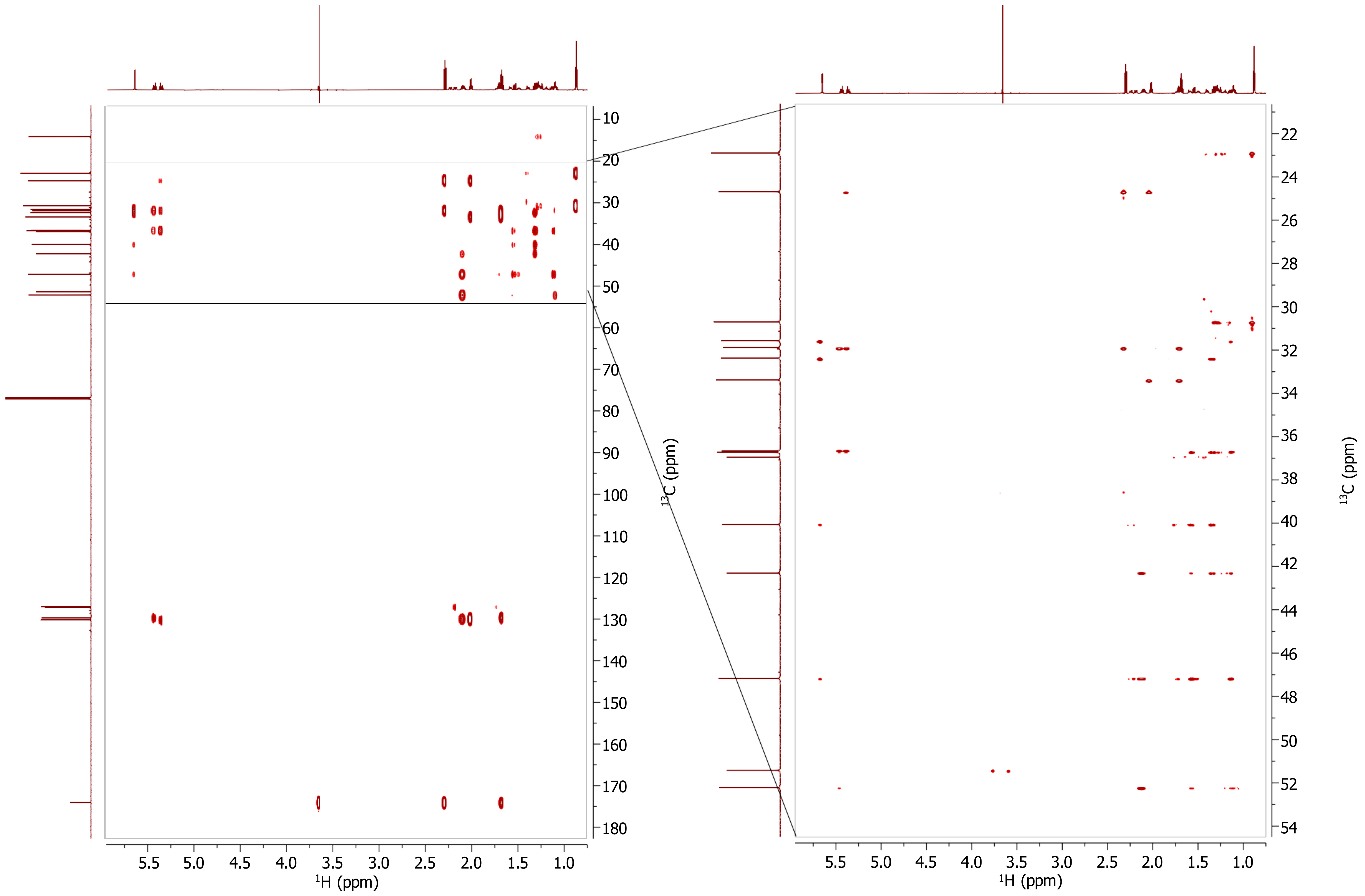

Figure S8: HMBC (left) and selective HMBC (right) of **7**

No	Atom Type	δ H (Multiplicity, *J*, nH)	δ C
**1**	CH	5.65 (m, 1H)	127.2
**2**	CH	5.65 (m, 1H)	127.3
**17**	CH	5.44 (dtt, 15.3,7.1,1.4 Hz, 1H)	130.2
**18**	CH	5.37	130.0
**25**	CH_3	3.66 (s, 3H)	51.4
**21**	CH_2	2.30 (t, 7.6 Hz, 2H)	33.4
**6'**	CH_2	2.24 (m, 1H)	31.6
**3'**	CH_2	2.19 (m, 1H)	32.4
**10**	CH_2	2.10 (m, 2H)	36.7
**19**	CH_2	2.02 (q, 7.1 Hz, 2H)	31.9
**3''**	CH_2	1.73 (m, 1H)	32.4
**6''**	CH_2	1.71 (m, 1H)	31.6
**20**	CH_2	1.69 (p, 7.5 Hz, 2H)	24.7
**8/27**	CH	1.59 (m, 1H)	42.3
**7'**	CH_2	1.55 (ddd, 12.2,7.3,2.9 Hz, 1H)	37.0
**4/12**	CH	1.50 (m, 1H)	40.1
**13'**	CH_2	1.40 (dddd, 12.7,10.0,5.1,5.1 Hz, 1H)	36.7
**7''**	CH_2	1.32 (ddd, 12.4,10.7,10.7 Hz, 1H)	37.0
**14'**	CH_2	1.29 (m, 1H)	30.7
**15**	CH_2	1.27 (m, 2H)	22.9
**14''**	CH_2	1.21 (m, 1H)	30.7
**13''**	CH_2	1.14 (dddd, 13.7,10.0,9.9,4.0 Hz, 1H)	36.7
**9/26**	CH	1.11 (m, 1H)	52.2
**5/11**	CH	1.10 (m, 1H)	47.3
**16**	CH_3	0.88 (t, 6.9 Hz, 3H)	14.2
**22**	C	-	174.2

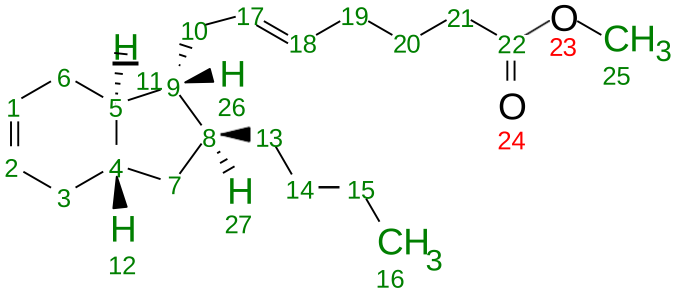

^{1}H NMR (850 MHz, $CDCl_3$) δ 5.68 – 5.63 (m, 2H), 5.44 (dtt, *J* = 15.3, 7.1, 1.4 Hz, 1H), 5.36 (dtt, *J* = 15.3, 6.7, 1.4 Hz, 1H), 3.66 (s, 3H), 2.30 (t, *J* = 7.6 Hz, 2H), 2.26 – 2.22 (m, 1H), 2.21 – 2.16 (m, 1H), 2.15 – 2.06 (m, 2H), 2.02 (q, *J* = 7.1 Hz, 2H), 1.76 – 1.71 (m, 1H), 1.72 – 1.69 (m, 1H), 1.69 (p, *J* = 7.5 Hz, 2H), 1.61 – 1.57 (m, 1H), 1.55 (ddd, *J* = 12.2, 7.3, 2.9 Hz, 1H), 1.53 – 1.47 (m, 1H), 1.40 (dddd, *J* = 12.7, 10.0, 5.1, 5.1 Hz, 1H), 1.32 (ddd, *J* = 12.4, 10.7, 10.7 Hz, 1H), 1.30 – 1.28 (m, 1H), 1.28 – 1.23 (m, 2H), 1.23 – 1.18 (m, 1H), 1.14 (dddd, *J* = 13.7, 10.0, 9.9, 4.0 Hz, 1H), 1.12 – 1.10 (m, 1H), 1.12 – 1.08 (m, 1H), 0.88 (t, *J* = 6.9 Hz, 3H).

^{13}C NMR (214 MHz, $CDCl_3$) δ 174.16, 130.29, 129.80, 127.25, 127.13, 52.23, 51.43, 47.18, 42.31, 40.07, 36.95, 36.73, 36.70, 33.41, 32.40, 31.92, 31.60, 30.73, 24.72, 22.93, 14.15.

Figure S9: Full assignment of **7**
